# Hot Deformation Behavior of a New Al–Mn–Sc Alloy

**DOI:** 10.3390/ma13010022

**Published:** 2019-12-19

**Authors:** Weiqi Kang, Yi Yang, Sheng Cao, Lei Li, Shewei Xin, Hao Wang, Zhiqiang Cao, Enquan Liang, Xi Zhang, Aijun Huang

**Affiliations:** 1 School of Materials Science and Engineering, University of Shanghai for Science and Technology, Shanghai 200093, China; weiqi.2ww@foxmail.com (W.K.); caozhiqiang@usst.edu.cn (Z.C.); 2 School of Materials, University of Manchester, Oxford Road, Manchester M13 9PL, UK; 3 Northwest Institute for Non-ferrous Metal Research, Xi’an 710016, China; mmstb207@163.com (L.L.); nwpu_xsw@126.com (S.X.); 4 Institute of Metal Research, Chinese Academy of Sciences, Shenyang 110016, China; haowang@imr.ac.cn; 5 Shanghai Aircraft Design and Research Institute, Shanghai 201210, China; liangenquan@comac.cc (E.L.); zhangxi@comac.cc (X.Z.); 6 Department of Materials Science and Engineering, Monash University, Clayton, VIC 3800, Australia; Aijun.huang@live.co.uk

**Keywords:** Al–Mn–Sc alloy, hot deformation, flow stress, processing map, dynamic recrystallization

## Abstract

The hot deformation behavior of a new Al–Mn–Sc alloy was investigated by hot compression conducted at temperatures from 330 to 490 °C and strain rates from 0.01 to 10 s^−1^. The hot deformation behavior and microstructure of the alloy were significantly affected by the deformation temperatures and strain rates. The peak flow stress decreased with increasing deformation temperatures and decreasing strain rates. According to the hot deformation behavior, the constitutive equation was established to describe the steady flow stress, and a hot processing map at 0.4 strain was obtained based on the dynamic material model and the Prasad instability standard, which can be used to evaluate the hot workability of the alloy. The developed hot processing diagram showed that the instability was more likely to occur in the higher Zener–Hollomon parameter region, and the optimal processing range was determined as 420–475 °C and 0.01–0.022 s^−1^, in which a stable flow and a higher power dissipation were achieved.

## 1. Introduction

Casting and wrought aluminum (Al) alloys have been widely used as structural materials in aerospace industries owing to their high specific strength (strength to weight ratio), excellent fatigue resistance, and good formability [1,2,3]. The strength mainly arises from precipitation strengthening achieved from aging treatment [4,5,6]. Recently, a new high strength Al–Mn–Sc alloy has been developed by Jia et al. [7] using selective laser melting (SLM). The supersaturated Mn and Sc significantly improve the mechanical property through solid solution strengthening of Mn and precipitation strengthening of nano-sized Al_3_Sc precipitates, which lead to a superior yield strength at 560 MPa and a good ductility at 18%. Such mechanical properties are attractive for aerospace industries. However, this Al–Mn–Sc alloy has only been studied in the additive manufactured condition, but has not been investigated in other forms like casting and wrought products.

After direct chill casting, Al ingots generally need various thermo-mechanical processing steps to obtain different types of semi-finished products. The microstructure of the material depends on the thermo-mechanical processing parameters, which also determine the quality of the formed part. Thus, it is necessary to understand the influence of deformation parameters on hot deformation behavior and microstructure in this Al–Mn–Sc alloy. In general, the isothermal compression test is an appropriate method to study the hot deformation characteristics of materials [8,9]. The relationships among the flow stress, strain, strain rate, and deformation temperature can be used to establish the hot deformation constitutive equation, and its hot activation energy can be calculated. The hot deformation behavior can be quantitatively described and applied to simulate the dynamic response of the material under specific loading conditions. In addition, the hot processing map is constructed to predict the plastic deformation mechanism and the unstable deformation domain in various deformation conditions, which provides insights into the optimization of thermo-mechanical processing. This method has been widely used in various alloys, such as Al alloys [8,9,10], Mg alloys [11,12,13], Ti alloys [14,15,16], and steel [17,18,19].

The present work aims to investigate the hot deformation behavior, to reveal the microstructure change, and to obtain the hot processing map of the casting Al–Mn–Sc alloy over wide temperature and strain rate ranges. The results will provide guides on its hot deformation processing and industrial applications.

## 2. Materials and Methods

The raw material (Table 1) used in this experiment was a casting ingot with a diameter of 150 mm. Cylindrical samples (φ10 × 15 mm) were sectioned by Electrical Discharge Machining (EDM) wire-cut from the ingot and then ground by SiC abrasive sandpaper before subjecting to isothermal hot deformation experiments conducted on a Gleeble-3800 system. According to the deformation conditions of aluminum alloy in normal industrial production, the test was carried out at temperatures from 330 to 490 °C and strain rates from 0.01 to 10 s^−1^. Graphite sheet was used as a lubricant between the compression plate and the sample to reduce friction. Before the compression test, the samples were solution treated at 500 °C for 5 min followed by gas quench in the Gleeble chamber. These samples were heated again to the testing target temperature at a ramping rate of 10 °C/s and held for 5 min to eliminate the thermal gradients before compression. Isothermal hot deformation experiments were conducted afterwards at various temperatures and strain rates. After a 60% deformation, the samples were gas-quenched to room temperature to freeze the microstructure after the hot deformation. The gas-quenched deformed samples were sectioned by EDM wire-cut along the axial direction, which is parallel to the compression direction. A standard metallographic sample preparation and etching by Keller’s solution (1 mL HF + 1.5 mL HCl + 2.5 mL HNO_3_ + 95 mL H_2_O) were carried out, and the microstructure characterization was conducted by using a Leica DMi8A light microscope (LM) and a FEI QUANTA 450 scanning electron microscope (SEM) equipped with an energy dispersive X-ray spectrometer (EDS) detector. The grain size was measured by ImageJ software.

## 3. Results and Discussion

### 3.1. Microstructure of As-Cast Al–Mn–Sc Alloy

**Figure 1:**
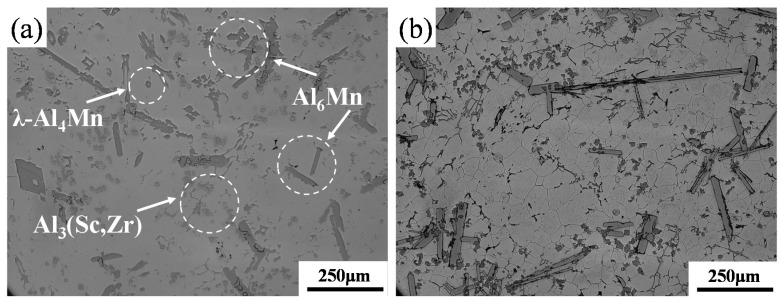
Light microscope (LM) images of the as-cast Al–Mn–Sc alloy of the (**a**) unetched and (**b**) etched sample revealing the grain boundaries.

Adding Mn element to aluminum alloy has a certain solid solution strengthening effect. In addition, the Al_6_Mn phase can hinder the growth of recrystallized grains, refine the grains, and improve the strength of the alloy. For Scandium (Sc) addition, the Al_3_Sc phase with an L12 structure can prevent recrystallization and promote fine grain strengthening and fine precipitation strengthening. Sc is considered to be the most effective alloying element for aluminum alloys. Al_3_Sc precipitates show small lattice mismatches and low interfacial energy in aluminum matrix, and the low diffusivity of Sc also helps to improve thermal stability [20]. The addition of Zr can further reduce the lattice mismatch, because Zr has a lower diffusivity, and can replace some Sc atoms to form a protective shell by segregating around the precipitate, thereby further enhancing the strengthening effect and thermal stability [21]. The as-cast microstructure of the Al–Mn–Sc alloy (Figure 1) is composed of intermetallic phases with different morphologies, including polygonal shaped phases, long lath-like phases, and small-sized hexagonal and square phases, distributed in the Al matrix. The etched samples revealed grain boundaries, as shown in Figure 1b, the matrix grains were equiaxed with an average size of approximately 30 µm according to image analysis.

**Figure 2:**
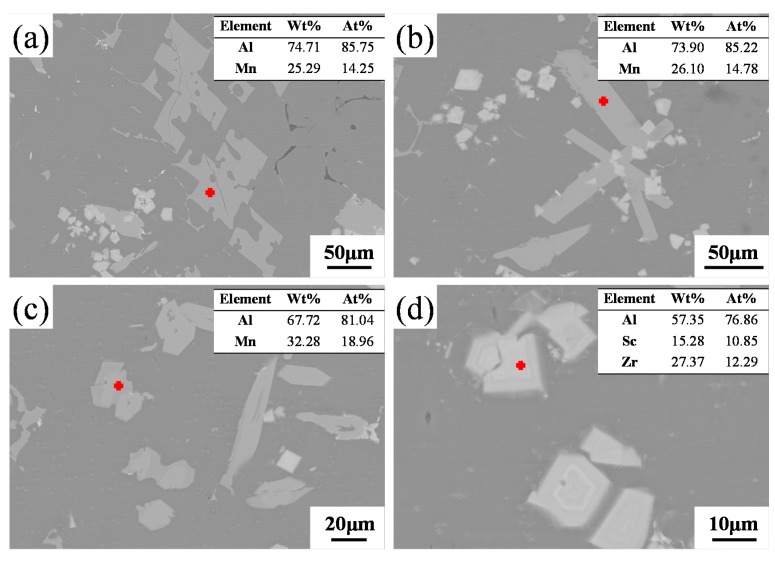
Scanning electron microscope (SEM) images and associated energy dispersive X-ray spectrometer (EDS) analyses for (**a**,**b**) Al_6_Mn phases with two different morphologies, (**c**) λ-Al_4_Mn phase with an irregular morphology, and (**d**) squared Al_3_(Sc, Zr) phase. The red crosses were the locations of EDS analyses in each image.

In order to identify these intermetallic phases, EDS analyses were applied. The larger sized polygonal and lath-like phases were enriched in Al with a small amount of Mn (Figure 2a,b), which suggested they were Al_6_Mn. The primary Al_6_Mn was in a polygonal shape and had sharp edges and corners, showing an obvious facet growth behavior. After intermetallic growth, the primary phases grew preferentially at the polygon corners, and a continuous slab shaped Ti_6_Mn was finally obtained. The EDS results revealed the hexagonal phases (Figure 2c) having a slightly higher Mn compared with other intermetallic phases (Figure 2a,b), which is similar to the λ-Al_4_Mn reported in a previous study [22]. The smaller-sized (~10 µm) square phases (Figure 2d) were enriched in Al, Sc, and Zr, which should be Al_3_(Sc,Zr), as Zr can replace some of the Sc atoms in the Al_3_Sc. The replaced Sc can segregate to the edge of the precipitates and form a protective shell [23].

### 3.2. Hot Deformation Behavior

In order to measure the validity of the thermal compression data, we verified the expansion coefficient *B* of the material; Equation (1) is as follows [24]:

(1) $$B=\frac{L_{0}d_{0}^{2}}{L_{f}d_{f}^{2}}$$

In Equation (1), *L*_0_ is the original height of the sample, *d_0_* is the original diameter of the sample, *L_f_* is the average height of the sample after compression (measured at the center axis of the cylinder and every 120° at the edge, and the average height is based on these four locations), and *d_f_* is the average diameter of the sample after compression (taken the average diameter at top, middile, and bottom heights). When *B* ≥ 0.9, the results of the thermal compression experiment are valid. After measurement and calculation, the thermal compression experimental data obtained by all samples are verified to be valid.

**Figure 3:**
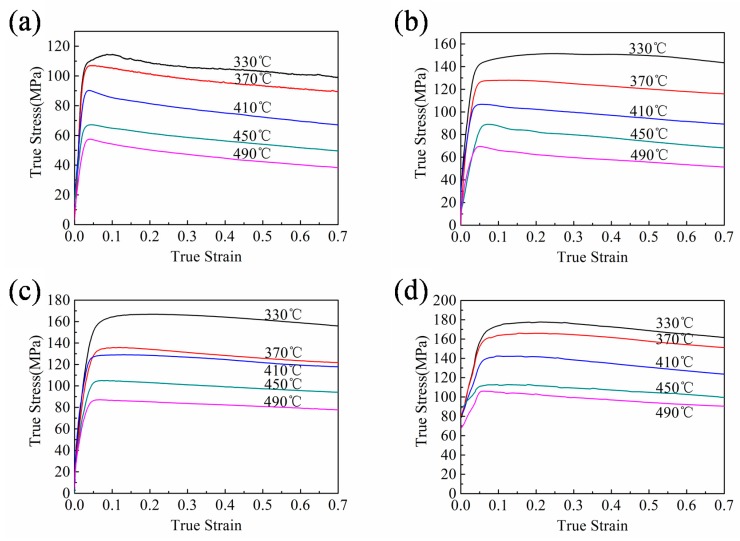
True stress–strain curves of the Al–Mn–Sc alloy deformed at various temperatures from 330 to 490 °C with different strain rates at (**a**) 0.01 s^−1^, (**b**) 0.1 s^−1^, (**c**) 1 s^−1^, and (**d**) 10 s^−1^.

On the basis of the true stress–strain curves of Al–Mn–Sc alloy compressed at different temperatures and strain rates (Figure 3), the flow behavior of the Al–Mn–Sc alloy was affected by the deformation temperature and strain rate. Work hardening caused by dislocation generation and entanglement resulted in an increase in flow stress at small strains. A further increase in strain led to a dynamic softening effect, which gradually overweighed the work hardening effect. As a result, the flow stress first increased to a peak and then slightly decreased with strains. In addition, the flow stress decreased substantially with increasing temperatures and reducing strain rates. This indicates the dynamic softening is more sufficient at high temperatures and low strain rates, which is consistent with the previous observations in other hot compressed Al alloys [25,26]. Dynamic softening, including dynamic recovery (DRV) or dynamic recrystallization (DRX), reduces the dislocation density in contrast to work hardening. A lower strain rate allows a longer time to accumulate the activation energy, which reduces the stress in turn.

### 3.3. Constitutive Equations

Deformation temperature and strain rate are important factors controlling the hot deformation flow stress. The hyperbolic sinusoidal constitutive equation in the Arrhenius model has been widely used to describe the complex relationships among flow stress, heat distortion temperature, and strain rate [8,9,10,11,12,13,14,15,16,17,18,19,27]. Sellars and McTegart proposed the use of a hyperbolic sine function including the thermal deformation activation energy *Q* and temperature *T* to describe the thermal activation behavior of the material. The relation among the strain rate, flow stress, and deformation temperature can be established by the following equation [28]:

(2) $$\dot{\varepsilon }=AF\left(\sigma \right)\exp\left(-Q/RT\right)$$

where $\dot{\varepsilon }$ is the strain rate, *A* is the structural factor, *F*(*σ*) is a function of stress, *σ* is the flow stress, *Q* is the activation energy, *R* is the gas constant, and *T* is the absolute temperature. At different stress conditions, *F*(*σ*) has the following three expressions:

(3) $$F\left(\sigma \right)=\{\begin{array}{l} \sigma^{n}\;\left(\alpha \sigma \leq 0.8\right)\\ \exp\left(\beta \sigma \right)\;(\alpha \sigma >0.8)\\ {\left[\sin h\left(\alpha \sigma \right)\right]}^{n}\;\left(for\;all\;\sigma \right) \end{array}$$

where *α* is the stress level parameter and *n* is the strain hardening index, *α = β/n*. *ασ* ≤ 0.8 represents a low stress level, and *ασ* > 0.8 represents a high stress level. Substituting different stress levels of Equation (3) into Equation (2) leads to Equations (4)–(6):

(4) $$\dot{\varepsilon }=A_{1}\sigma^{n_{1}}$$

(5) $$\dot{\varepsilon }=A_{2}\exp\left(\beta \sigma \right)$$

(6) $$\dot{\varepsilon }=A{\left[\sin h\left(\alpha \sigma \right)\right]}^{n}\exp\left(-Q/RT\right)$$

In order to determine the constant terms in Equations (4) and (5), the natural logarithm is applied on both sides of the equation, and the following equations can be obtained:

(7) $$\ln\dot{\varepsilon }\;=\;\ln A_{1}\;+\;n_{1}\ln\sigma$$

(8) $$\ln\dot{\varepsilon }\;=\;\ln A_{2}\;+\;\beta \sigma$$

**Figure 4:**
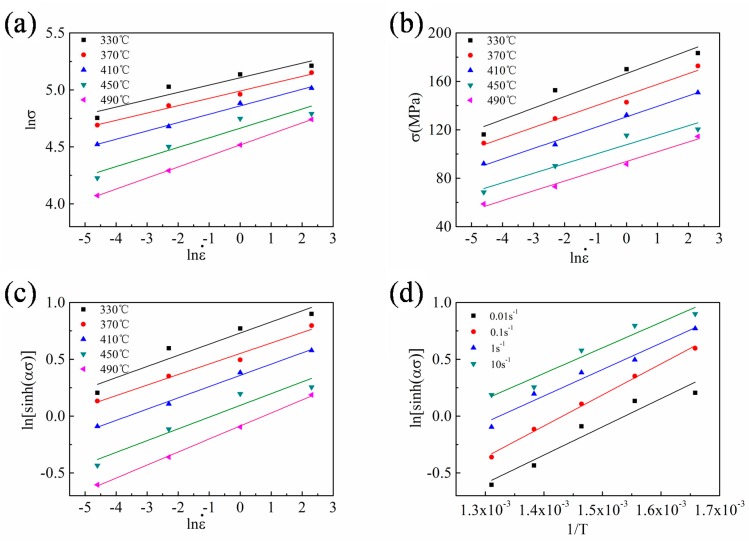
Linear curve fittings of (**a**) ln$\dot{\varepsilon }$ vs. ln*σ*, (**b**) ln$\dot{\varepsilon }$ vs. *σ*, (**c**) ln$\dot{\varepsilon }$ vs. ln[sinh(*ασ*)], and (**d**) 1/*T* vs. ln[sinh(*ασ*)].

On the basis of Equations (7) and (8), the relationship between the stress and strain rate (Figure 4) can be obtained by plotting using measured peak stress (Table 2). The curve fitting was conducted by a linear least-squares regression. The average slopes of all the fitted lines are the constant *n*_1_ and *β*, respectively (Figure 4a,b). The obtained *n*_1_ is 13.058, and *β* is 0.116. Hence, *α* is calculated at 0.009.

By applying Equation (6) to all stress levels, Equation (8) can be obtained by taking the natural logarithm:

(9) $$\ln\dot{\varepsilon }=n_{2}\ln\left[\sinh\left(\alpha \sigma \right)\right]+\ln A-Q/RT$$

The following equation of the hot activation energy *Q* can be obtained from Equation (9):

(10) $$\frac{Q}{T}=R\left\{\ln A-\ln\dot{\varepsilon }+n_{2}\ln\left[\sinh\left(\alpha \sigma \right)\right]\right\}$$

At a certain strain and strain rate, Equation (11) can be derived from Equation (10):

(11) $$\frac{Q}{Rn_{2}}=\left\{\frac{\partial \ln\left[\sinh\left(\alpha \sigma \right)\right]}{\partial \left(1/T\right)}\right\}$$

In Equation (9), *n* is the average slope of the linear relationship between $\ln\dot{\varepsilon }$ and ln[sinh(*ασ*)], and *Q/Rn* is the average slope of the linear relationship between ln[sinh(*ασ*)] and (1/*T*) in Equation (11). As shown in the Figure 4c,d, the mean values of these two slopes are 9.851 and 2449.059 respectively. As we know the value of *n*_2_ and *R*, the hot deformation activation energy *Q* of the Al–Mn–Sc alloy is determined at 200.581 kJ·mol^−1^. According to Zener and Hollomon [27,29], the strain rate of high temperature plastic deformation is controlled by the heat activation process, and the relationship between strain rate and temperature can be expressed by the *Z* parameter, which is a temperature compensated strain rate factor. Hence, Equation (2) can be further derived to Equation (12):

(12) $$Z=\dot{\varepsilon }\exp\left(\frac{Q}{RT}\right)=A{\left[\sin h\left(\alpha \sigma \right)\right]}^{n_{3}}$$

By applying the natural logarithm in Equation (12), we can obtain the following equation:

(13) $$\ln Z=\ln\dot{\varepsilon }+\frac{Q}{RT}=n_{3}\ln\left[\sinh\left(\alpha \sigma \right)\right]+\ln A$$

**Figure 5:**
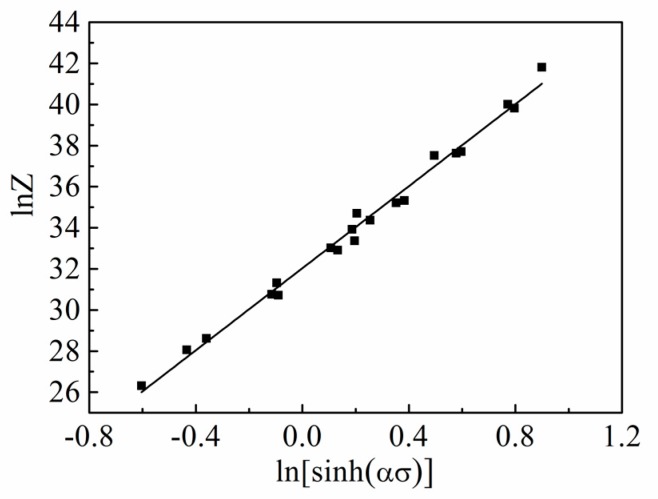
Linear relationship between ln[sinh(*ασ*)] and the Zener–Hollomon parameter, ln*Z*.

By substituting the values of *T* and $\ln\dot{\varepsilon }$ into Equation (13), the value of the Zener–Hollomon parameter (ln*Z*) at different temperatures and strain rates can be obtained. As shown in Figure 5, it is worth noting that the value change trend of ln*Z* is the same as the flow stress, and increases as the deformation temperature decreases or the strain rate increases. The slope at 9.978 is computed between ln*Z* and ln[sinh(*ασ*)], which leads to the structural factor *A* at 8.171 × 10^13^. In summary, all constant values are determined above. The constitutive equation for the hot compressed Al–Mn–Sc alloy can then be presented in Equation (14).

(14) $$\dot{\varepsilon }=8.171\times 10^{13}{\left[\sin h\left(0.009\sigma \right)\right]}^{9.987}\exp\left(-\frac{200581}{RT}\right)$$

In general, the activation energy *Q* is closely related to the thermodynamic mechanism of dislocation movement and can reflect the processability of the material. It is thus meaningful to understand the effect of processing parameters on activation energy.

According to Equations (9) and (11), the activation energies obtained under various deformation conditions are shown in Table 3.

As shown in Table 3, the deformation activation energy of the Al–Mn–Sc alloy decreases with increasing temperatures and strain rates. When the hot working conditions are changed in a wide range, the obtained deformation activation energy is also in a large range, which indicates that the alloy is sensitive to hot working deformation conditions.

First, the activation energy decreases with increasing strain rates. This phenomenon should be related to dislocation movement. The external stress increases with increasing strain rates, and the shear stress applied in the dislocation sliding direction also increases [30]. Therefore, dislocation motion can be activated easily at a higher stress and lower activation barrier condition [31].

Second, the activation energy decreases with increasing deformation temperatures owing to the effect of dislocation density. At higher temperatures, the rearrangement of dislocations during DRV and the formation and growth of recrystallized grains during DRX will be promoted, leading to a reduction in dislocation density. Therefore, as the deformation temperature increases, the resistance to dislocation movement decreases.

### 3.4. Processing Map

The thermo-mechanical processing maps (PMs) were extensively used to describe the microstructure evolution and establish the processing window, which provide guides for the industrial manufacturing such as rolling, extrusion, and forging [10,14,32,33]. PMs can be obtained by the dissipation power diagram and the processing instability diagram. On the basis of the principles of large plastic deformation continuum mechanics, physical system simulation, and irreversible thermodynamics theory, Prasad et al. established a dynamic material model (DMM), which regards the hot deformation process as a closed thermodynamic system [34].

During thermo-mechanical processing, the energy *P* obtained by the material per unit volume within a certain time can be divided into two parts according to the report by Prasad et al. [34]: (1) The energy consumed by plastic deformation is represented by *G*. Most of *G* is converted into heat, and a small portion is stored as crystal defect energy. (2) The energy consumed by the microstructure evolution during hot deformation is *J*, which represents the evolution of the microstructure during the deformation process, such as DRV, DRX, internal cracks (voids formation and wedge cracks), dislocations, growth of grains and precipitates under dynamic conditions, spheroidization of needle-like structures, phase transitions [35], and so on. Therefore, the total energy *P* can be expressed as follows:

(15) $$P=G+J=\int_{0}^{\dot{\varepsilon }}\sigma d\dot{\varepsilon }+\int_{0}^{\sigma }\dot{\varepsilon }d\sigma =\sigma \dot{\varepsilon }$$

For most pure metal or low alloy materials, the energy distribution relationship between *G* and *J* satisfies Equation (16) when the temperature and strain rate are constant:

(16) $$m=\frac{\partial J}{\partial G}=\frac{\dot{\varepsilon }\partial \sigma }{\sigma \partial \dot{\varepsilon }}=\frac{\partial \ln\sigma }{\partial \ln\dot{\varepsilon }}$$

where m is the strain rate sensitivity factor and is independent to strain. For the condition of the ideal linear dissipation (*m* = 1), *J* has the maximum value at *J_max_* = *P*/2. The ratio between *J* and *J_max_* was determined by the dissipation efficiency factor (*ƞ*) [34]:

(17) $$\eta =\frac{J}{J_{\max}}=\frac{P-G}{P/2}=\frac{2m}{1+m}$$

where *ƞ* represents a dimensionless parameter that describes the ratio between the energy consumed by the microstructure evolution and the total energy consumed by a linear hot deformation. *ƞ* is also termed as the microstructure trace. Domains with high dissipation efficiency factors in PMs indicate the formation of special structures or occurrence of softening behaviors, such as DRX and DRV, and possible local deformation instability. Therefore, the unstable region of the material in hot deformation must be identified in order to determine a suitable processing window [34,35]. On the basis of the principle of irreversible thermodynamics of large plastic deformation proposed by Prasad, the instability criterion is established as follows:

(18) $$\frac{dJ}{d\dot{\varepsilon }}<\frac{J}{\dot{\varepsilon }}$$

(19) $$\xi \left(\dot{\varepsilon }\right)=\frac{\partial \ln\left(\frac{m}{m+1}\right)}{\partial \ln\dot{\varepsilon }}+m<0$$

**Figure 6:**
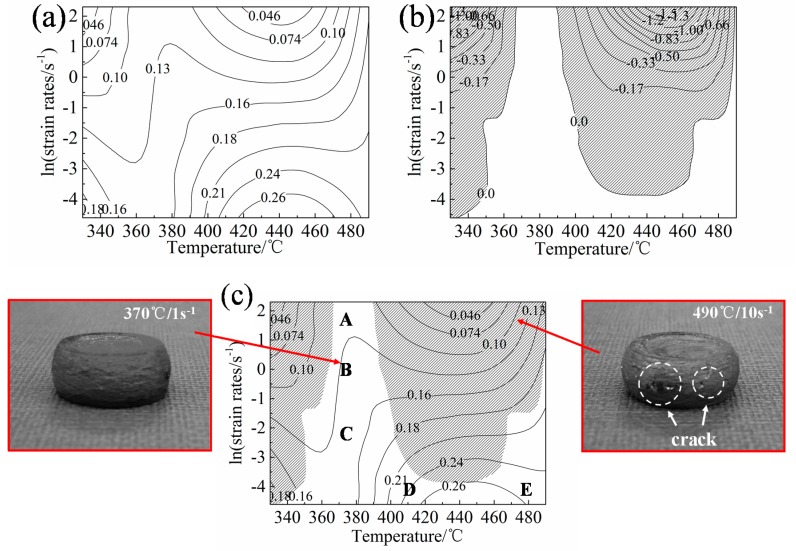
Power dissipation efficiency map (**a**), instability map (**b**), and processing map (**c**) of Al–Mn–Sc alloy at *ɛ* = 0.4.

The values of *m,* *η*, and *ξ* were calculated by cubic spline interpolation. The equivalent maps of the dissipated power *η* and the instability factor *ξ* are plotted in Figure 6a,b, respectively.

A domain with a higher dissipation efficiency factor indicates that more energy is dissipated in microstructure evolution, which is preferred as the processing window for hot deformation. Figure 6a shows a contour map of *η* at *ɛ* = 0.4. With the temperatures at 420–475 °C and the strain rate at 0.01–0.022 s^−1^, the dissipated power increases to the maximum value of 26%. Figure 6b shows the instability map of the Al–Mn–Sc alloy at *ɛ* = 0.4, and the shaded domains (the negative instability factor) represents the unstable windows. When the deformation temperature is low and the strain rate is large (i.e., a higher *Z* parameter), the instable deformation is more likely to occur. Hot processing in these parameters is prone to introduce defects and is not effective in facilitating the recrystallization. A suitable processing window should be the rest region outside the regions mentioned above.

Figure 6c is the PM generated by superimposing the dissipated power and the flow instability diagrams when the strain is fixed at 0.4. Comparing the samples under the two deformation conditions of 370 °C/1 s^−1^ (stable region) and 490 °C/10 s^−1^ (unstable region) in Figure 6c, it is apparent that severe cracking happened in 490 °C/10 s^−1^.

The peak domain (26%) with deformation temperatures and strain rates at 420–475 °C and 0.01–0.022 s^−1^ is the most suitable processing window. The strain rate is low, but the deformation temperature is high, and there should be enough driving force to promote the dynamic recovery or dynamic recrystallization to optimize the microstructure. Although the dissipated power value is low at 370 °C, regions A and B in Figure 6c are also interesting by considering that most conventional forming processes operate with forming rates significantly above 1s^−1^.

### 3.5. Microstructure Analysis

**Figure 7:**
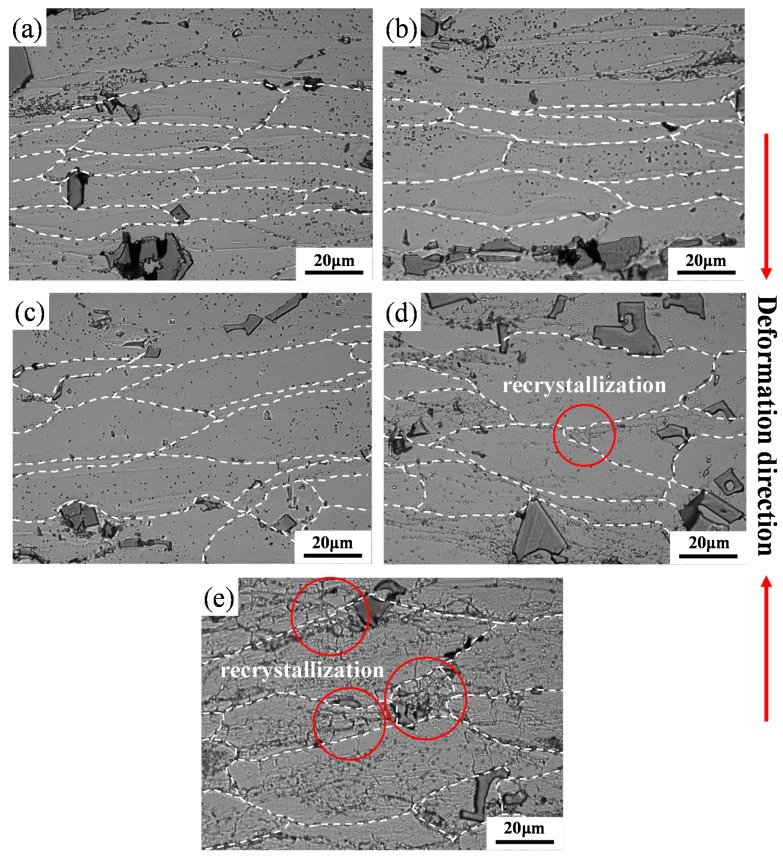
LM images of the Al–Mn–Sc alloy deformed at (**a**) 370 °C and 10 s^−1^, (**b**) 370 °C and 1 s^−1^, (**c**) 370 °C and 0.1 s^−1^, (**d**) 410 °C and 0.01 s^−1^, and (**e**) 490 °C and 0.01 s^−1^.

Many studies have shown that the microstructure after hot deformation is closely related to the Zener–Hollomon (*Z*) parameter [8,36,37,38,39]. The *Z* parameter was used to evaluate the hot deformation behaviours, and a larger ln*Z* corresponds to a higher strain rate or a lower deformation temperature. In order to further verify the feasibility of the hot processing map and understand the deformation mechanism, the deformed microstructures processed at the five regions (A) to (E) in Figure 6c are presented in Figure 7, and the corresponding ln*Z* are listed in Table 4. Figure 7 shows that grains are aligned in the transverse direction and intermetallic compounds are distributed in the matrix. As shown in Table 4, the ln*Z* value decreases with reducing deformation rates when the deformation temperature is 370 °C. Most of the grain boundaries remain straight, and DRV is the main softening mechanism in Figure 7a–c. The dislocations in this process change from the mixed arrangement of high-energy states to the regular arrangement of low-energy states, forming vertically arranged dislocation walls [8,33]. The strain rates of Figure 7a,d, and e are fixed at 0.01 s^−1^, and the value of ln*Z* decreases with an increasing deformation temperature, as shown in Table 4. The grain boundary is no longer straight and becomes relatively curved at high deformation temperatures. Moreover, some small grains appear at grain boundaries in sample deformed at 490 °C (Figure 7e), which are preferred locations for recrystallization [40]. Therefore, in these cases, DRX is the main mechanism for the hot deformation. In Figure 7e, the value of ln*Z* is the smallest with the deformation temperature at 490 °C and strain rate at 0.01 s^−1^, which leads to a substantially increased DRX and is consistent with the findings in previous studies [39,40,41]. DRX is beneficial for the hot deformation process, which provides a stable flow and results in a good processability. A high temperature accelerates the diffusion of atoms and promotes the microstructure change of materials. A reduced dislocation density achieved by either DRV or DRX is able to compensate the work hardening effect, which leads to a steady flow in thermo-mechanical processing. In addition, a low strain rate provides sufficient time for microstructural evolution during plastic deformation. Thus, high deformation temperature and low strain rates are favorable to achieve a steady-state deformation, and a processing window is proposed at 420–475 °C/0.01–0.022 s^−1^ for the new Al–Mn–Sc alloy.

## 4. Conclusions

Hot compression tests were conducted at 330–490 °C and 0.01–10 s^−1^ to study the hot deformation behavior and determine the processing window of a new Al–Mn–Sc alloy. The following conclusions were reached.

(1) The dynamic softening is sufficient at higher temperatures and lower strain rates. In general, the flow stress decreases with increasing deformation temperatures and decreasing strain rates. The deformation behavior satisfies the hyperbolic sinusoidal constitutive law with an activation energy *Q* at 200.581 kJ·mol^−1^ and a structural factor *A* at 8.171 × 10^13^.
(2) On the basis of DMM, a hot processing map is established at a 0.4 strain. The optimal processing range is between 420–475 °C and 0.01–0.022 s^−1^, in which a stable flow and a high power dissipation are achieved.
(3) In hot deformation, a high ln*Z* (low deformation temperatures and high strain rates) suggests a dynamic recovery dominated mechanism and a low ln*Z* indicates a dynamic recrystallization dominated mechanism.

## Figures and Tables

**Table 1:** Composition of the studied Al–Mn–Sc alloy (wt.%).

Mn	Mg	Sc	Zr	Si	Fe	Al
4.3–4.7	1.4–1.6	0.65–0.85	0.7–0.8	<0.1	<0.1	Bal

**Table 2:** Peak stress (MPa) values of Al–Mn–Sc alloy samples deformed at different conditions.

Strain Rate/s^−1^	Temperature/°C				
	330	370	410	450	490
0.01	116	110	92	68	59
0.1	153	129	108	90	73
1	170	143	132	115	92
10	183	173	151	120	115

**Table 3:** The activation barriers *Q* (kJ·mol^−1^) at different conditions in the isothermal deformation of Al–Mn–Sc alloy.

Strain Rate/s^−1^	Temperature/°C				
	330	370	410	450	490
0.01	210.439	222.745	208.569	199.904	180.28
0.1	231.688	245.236	229.629	220.089	198.486
1	197.168	208.697	195.415	187.297	168.913
10	190.941	202.106	189.244	181.382	163.578

**Table 4:** ln*Z* values of Al–Mn–Sc alloy samples deformed at different regions in the PM in Figure 6c.

Regions in PM	Temperature/°C	Strain Rate/s^−1^	Ln*Z*
A	370	10	39.823
B	370	1	37.520
C	370	0.1	35.217
D	410	0.01	30.718
E	490	0.01	26.317
